# Sleep and Ultramarathon: Exploring Patterns, Strategies, and Repercussions of 1,154 Mountain Ultramarathons Finishers

**DOI:** 10.1186/s40798-024-00704-w

**Published:** 2024-04-08

**Authors:** Anthony Kishi, Guillaume Y Millet, Matthieu Desplan, Bruno Lemarchand, Nicolas Bouscaren

**Affiliations:** 1 Unité Fonctionnelle de Médecine du Sport, CHU de la Réunion, Site Hôpital de Saint-Pierre, BP 350, Saint-Pierre, 97448 France; 2 Inter-university Laboratory of Human Movement Biology, Univ Lyon, UJM-Saint-Etienne, Saint-Etienne, 7424, F-42023 EA France; 3 Institut Universitaire de France (IUF), Paris, France; 4 Be Sports Clinic, Centre Médical Médimarien, Schaerbeek, 1030 Belgique; 5 Service de santé Publique et soutien à la recherche, INSERM CIC 1410, CHU Réunion, Saint Pierre, France

**Keywords:** Sleep deprivation, Mountain ultramarathon, Sleep pattern, Vigilance, Napping

## Abstract

**Background:**

Sleep and physical performance are strongly related and mutually influence each other. Athletes, particularly in disciplines like offshore sailing and ultra-endurance sports, often suffer from sleep deprivation due to factors like irregular training times, travel, and the extended duration of events like 100-mile mountain races. Despite growing interest in sleep’s role in sports science, few studies have specifically investigated the sleep patterns of ultramarathon runners. This study aimed to investigate sleep patterns and sleep management strategies in ultramarathons, and the repercussions of sleep deprivation during and after races.

**Methods:**

This cross-sectional study using e-survey was conducted on 1154 runners from two ultramarathons (a 165 km race with 9,576 m positive elevation; 2018 finish time [23:18:48–66:04:00], and a 111 km race with 6,433 m elevation; [15:34:56 − 41:54:16]).

**Results:**

The results revealed that 58% of the runners reported implementing sleep management strategies before or during the race. Most runners began the race with some level of sleep debt (-50 min a week before the race). During the races, 77% of runners slept, with the cumulative sleep duration varying based on race duration and the number of nights spent on the race (76 min at 165 km and 27 min at 111 km). Short naps lasting less than 30 min were the most popular strategy. The prevalence of symptoms attributed to sleep deprivation during the race was high (80%), with reported falls and hallucinations. After the race, runners reported recovering a normal state of wakefulness relatively quickly (within two days); 22% believed that sleep deprivation during the race increased the risk of accidents in everyday life.

**Conclusion:**

This study provides valuable insights into sleep patterns and strategies in ultramarathon running and emphasizes the importance of adequate sleep management for performance and post-race recovery.

**Supplementary Information:**

The online version contains supplementary material available at 10.1186/s40798-024-00704-w.

## Key Points

- On average, ultramarathoners start their race with a cumulative total sleep debt of -50 min over the seven days preceding the race compared to their usual weekly sleep duration.
- 77% of runners declared having done at least one nap during the races, yet the individual cumulative sleep duration was relatively low:76 min on a 165 km (finish time 49.9 ± 7.9 h) vs. 27 min on a 111 km (finish time 33.6 ± 5.3 h).
- The nap duration during the races was short (82% lasted less than 30 min) and occurred mainly during the second part of the night.
- 80% of runners reported at least one symptom attributed to sleep deprivation during the race. Those who increased their average daily sleep deprivation the week before the race reported a lower prevalence of falls attributed to sleep deprivation.
- Runners recovered from a normal state of wakefulness relatively quickly (within two days), but 22% believed that sleep deprivation during the race increased the risk of accidents in everyday life.

**Supplementary Information:**

The online version contains supplementary material available at 10.1186/s40798-024-00704-w.

## Background

Sleep and physical performance are strongly related and mutually influence each other [1, 2]. Sleep deprivation of 24–30 h has been shown to lower reaction time [3] and impair cognitive performance [3–5], and reduce muscle glycogen stores [6]. Conversely, sleep extension strategies implemented before sleep loss have been found to enhance performance by preventing decline in cognitive performance [7], improving sustained attention, reducing sleep pressure [8], and enhancing motor performance [9]. Increasing the total sleep time over a relatively short period may mitigate the negative effects of sleep deprivation and provide direct benefits across disciplines [10–12]. In addition to performance, sleep deprivation has notable effects on recovery processes [13]. For example, research has shown that one night of partial sleep deprivation impaired recovery from a high-intensity interval training session among cyclists [14]. Additionally, total sleep deprivation following eccentric exercise-induced muscle damage alters inflammatory and hormonal responses without impeding muscle strength recovery [15].

Athletes often experience partial sleep impairment due to early morning and evening training sessions, traveling across time zones for competitions, and poor sleep quality during heavy training before and after competitions [16, 17]. Certain sports disciplines, such as offshore sailing or ultra-endurance sports, inherently involve total sleep deprivation for one or several nights owing to their prolonged duration. The 100-mile mountain races, such as the Western States Endurance Race® and the Ultra-Trail du Mont Blanc®, typically require 20–24 h for top runners and up to 48 h for most participants to complete. Consequently, long-distance races can leave the slowest athletes sleep-deprived for several consecutive nights. Although sleep has gained increasing attention in sports science over the past decade, only a limited number of studies have examined sleep patterns of ultramarathon runners [2, 18–21].

Research has suggested that the sleep duration of ultramarathon runners on weekdays (6–8 h per night) is comparable to that of the general and athletic populations, with a lower prevalence of sleep disorder-related symptoms [2]. Previous research has indicated that 48–88% of ultramarathon runners employ sleep extension strategies before races, predominantly through napping (35–41%) or increasing sleep time during the nights preceding the race (33–55%) [2, 18, 21]. Runners who increased their sleep duration the night before the race completed it at a faster pace [21]. Furthermore, 69–72% of Ultra-Trail du Mont Blanc® (UTMB®) finishers reported no sleep during the race [21, 22]. As expected, these runners achieved faster race times than those who slept, and drowsiness during the race positively correlated with race duration [21]. Although not all athletes sleep during ultramarathon races (e.g., 100-mile event), those who do typically rely upon brief naps [18].

Therefore, this descriptive study aimed to further investigate sleep strategies in ultramarathon runners using a larger dataset and longer race durations, and to provide new insights into the effects of race duration on sleep. The primary objective of this study was to describe the sleep habits of ultramarathon runners and their sleep management strategies before, during, and after race. The secondary objective was to provide original information on sleep management comparison during two ultramarathons at different distances (165 km vs. 111 km). Finally, this study aimed to investigate the physical and psychological repercussions of sleep deprivation during and after races.

## Methods

### Study Design and Setting

This cross-sectional study, conducted during the 2018 Grand Raid de La Reunion, utilized an anonymous e-survey questionnaire (Google Form Survey). The study was approved by the Clinical Research and Innovation Department of Reunion University Hospital (reference 3,408,030,719, 12/07/2018) and the Grand Raid Reunion medical board. All the runners gave their consent to participate. The Grand Raid de La Reunion Island attracts over 6,000 runners annually, who participate in one of the three races. This study specifically targeted participants of the two longest races: La Diagonale des Fous (DDF:165 km; 9,576 m positive elevation in semi-autonomy; 2018 finish time [23:18:48–66:04:00]), which crosses the island from south to north, and Le Trail de Bourbon (TDB:111 km; 6,433 m elevation; [15:34:56 − 41:54:16]).

### Survey Content

The survey comprised several sections: demographic data, training characteristics, ultramarathon experience, usual sleep profile (bedtime, wake-up time, average daily sleep duration (ADSD), and desired ideal daily sleep duration), and circadian typology (morning, evening, and neither type). Additionally, the participants were asked to complete retrospectively a sleep diary covering the week preceding the race period, providing information on bedtime, sleep quality, and hours of sleep. The difference between the sleep duration for each day of the week before the race and the ADSD was calculated to determine sleep debt. Total sleep debt for the week preceding the race was obtained by summing the estimated daily sleep debt for each day compared to ADSD. The survey also included questions regarding the sleep strategies implemented before, during, and after the race.

In the race-specific section of the survey, runners were asked to describe their nap and sleep schedules during the race, the conditions under which they occurred, and the potential repercussions of sleep deprivation (e.g., falls, injuries, hallucinations, and endangerment). Sleep patterns during the recovery period were documented along with the physical and psychological effects of sleep deprivation on daily living. The research team ensured that no personal information or emails were collected. The English version of the survey is available in the Supplementary Material.

### Data Collection Methodology and Survey Implementation

A single questionnaire was sent post-race to retrospectively capture data from the pre-race period, during the race, and after the race. To ensure the accuracy and reliability of our retrospective sleep data collection and to mitigate potential recall biases, participants were briefed through an initial email information about the importance of being observant of their sleep patterns in the week leading up to the race. They were encouraged, when possible, to note key sleep-related information, including daily sleep duration (night and naps) the week before the race, sleep difficulties in the 24 h preceding the race, naps during the race (duration, location), symptoms of sleep deprivation, and recovery period details. This preparatory guidance aimed to enhance the precision of the data retrospectively captured in the survey. With the race occurring from October 18 to 21, the questionnaire was distributed on November 2 to assess the recovery period with sufficient hindsight. Three follow-up reminders were sent; 82% of the runners responded within the first seven days, with the last participant replying on December 16.

### Variables and Statistics Methods

Participants were categorized into three groups based on the number of nights spent during the race: one, two, or three nights. The definition of “spending a night in the race” was established by the authors as runners remaining in the race for at least 4 h between 6:00 PM (sunset time) and 6:00 AM (sunrise time). The finish times of the runners were collected from the official race results and are presented in deciles for each race.

Due to the limited number of non-finishers (*n* = 9), only data from race-finishers were analyzed. Bivariate analyses were conducted using the Pearson Chi-squared test or Fisher’s exact test for categorical variables and Student’s t-test or Wilcoxon signed-rank test for quantitative variables, as appropriate. Paired t-tests were performed to compare ADSD seven days before the race with the usual ADSD. Additionally, a pairwise correlation coefficient was calculated to assess the relationship between sleep duration and race-finish time. The significance level was set at *p* < 0.05. All statistical analyses were performed using Stata V13 software (StataCorp LP, College Station, TX, USA).

## Results

### Participants

Among 3,827 runners eligible for the study, 1,163 (30.4%) responded to the survey. Of these, 1,154 subjects were finishers (882 runners for *DDF* and 275 for *TDB)*, 85.7% were men, with a mean age of 42.8 ± 9.0 years. The sex ratio and age distribution of the participants were representative of overall race starters. Table 1 presents the participants’ demographic and training characteristics. The average finish time was 49.9 ± 7.9 h for DDF and 33.6 ± 5.3 h for TDB.

**Table 1:** Characteristics of study participants total and according to the race distance

	Total (*n* = 1154)	La Diagonale Des Fous (*n* = 882)	Le Trail De Bourbon (*n* = 272)
**Demographic characteristics**			
**Gender**, n (%)			
Male	989 (85.7)	774 (87.8)	215 (79.0)
Female	165 (14.3)	108 (12.2)	57 (21.0)
**Age** (years)	42.8 ± 9.0	43.1 ± 8.9	41.9 ± 9.1
**Place of residence**, n (%)			
Reunion Island	459 (39.8)	284 (32.2)	175 (64.4)
Metropolitan France	608 (52.7)	529 (60.0)	79 (29.0)
Another country	87 (7.5)	69 (7.8)	18 (6.6)
**Training characteristics**			
**Weekly training duration** (hours)	8.3 ± 4.0	8.7 ± 4.1	7.2 ± 3.5
**Experience in trail running** (years)	7.2 ± 5.2	7.5 ± 5.2	6.0 ± 5.0
**Number of ultra-race participation**	6.2 ± 6.5	6.3 ± 6.6	5.6 ± 5.8
**First ultra-trail race longer than 80 km** n (%)	267 (23.1)	132 (15.0)	135 (49.6)

Data are presented as n (%) or mean ± standard deviation

### Sleep Characteristics, Circadian Typology, and Sleep Deprivation Symptoms

ADSD reported by participants was 7.5 ± 1.0 h, while the desired ideal daily sleep duration was 8.0 ± 1.0 h. Working at night was reported by 15.0% of participants. Approximately 19.3% of the participants reported a history of sleep disorder-related symptoms, including regular insomnia (12.0%), sleep apnea (5.3%), and sleep disorders linked to depressive syndrome (2.0%). Sleeping pills were consumed by 4.7% of the runners.

### Sleep Strategy

Implementation of at least one sleep management strategy in preparation for the race was expressed by 58.3% of the runners [61.7% for *DDF* and 47.4% for *TDB* (*p* < 0.001)]. These strategies were planned for the pre-race period by 39.9% of the participants [40.7% for *DDF* and 37.1% for *TDB* (*p* = 0.29)] and 41.9% for race [46.9% for *DDF* and 25.4% for *TDB* (*p* < 0.001)]. Notably, 18.3% of the participants reported practicing training while deliberately depriving themselves of sleep before the race, but this strategy was not associated with a better finish time (*p* = 0.66).

### Runners’ Sleep before the Race

Table 2 presents the sleep modifications reported by runners during the week leading up to their race. Overall, 61% of the runners experienced at least one sleep modification compared to their normal sleep patterns. Among them, 54.9% reported an increase in ADSD, 5.9% experienced a modification in their sleeping schedule (bedtime or wake-up time), and 1.0% reported a reduction in ADSD. Additionally, 46.4% of runners experienced one night of partial sleep disturbance in the seven days preceding the races [50.2% for *DDF* and 33.8% for *TDB* (*p* < 0.001)]. However, runners who reported an increase in average sleep duration (*p* = 0.37) or changes in their sleeping schedules (*p* = 0.21) did not finish the race faster than the others did.

**Table 2:** Categories of sleep duration adaptation Prior to Ultramarathons the week before the event

	Total (*n* = 1154)	La Diagonale Des Fous-DDF (*n* = 882)	Le Trail De Bourbon-TDB (*n* = 272)	*p*
**Runners with increased in average sleep duration**, n (%)	**633 (54.9)**	**500 (56.7)**	**133 (48.9)**	**0.02**
Increase of naps duration alone	133 (11.5)	103 (11.7)	30 (11.0)	
Increase of sleep duration at night alone	281 (24.4)	216 (24.5)	65 (23.9)	
increase of both	219 (19.0)	181 (20.5)	38 (14.0)	
**Runners without change in average sleep duration but with changes in sleep schedules**, n (%)	**68 (5.9)**	**53 (6.0)**	**15 (5.5)**	0.76
Earlier bedtime and earlier wake-up	46 (4.0)	36 (4.1)	10 (3.7)	
Later bedtime and later wake-up	22 (1.9)	17 (1.9)	5 (1.8)	
**Runners with reduction in average sleep duration**, n (%)	**12 (1.0)**	**9 (1.0)**	**3 (1.1)**	1

Data are presented as number of runners and percentage for each category. All *p* values refer to comparisons between *DDF* and *TDB*

**Fig. 1:**
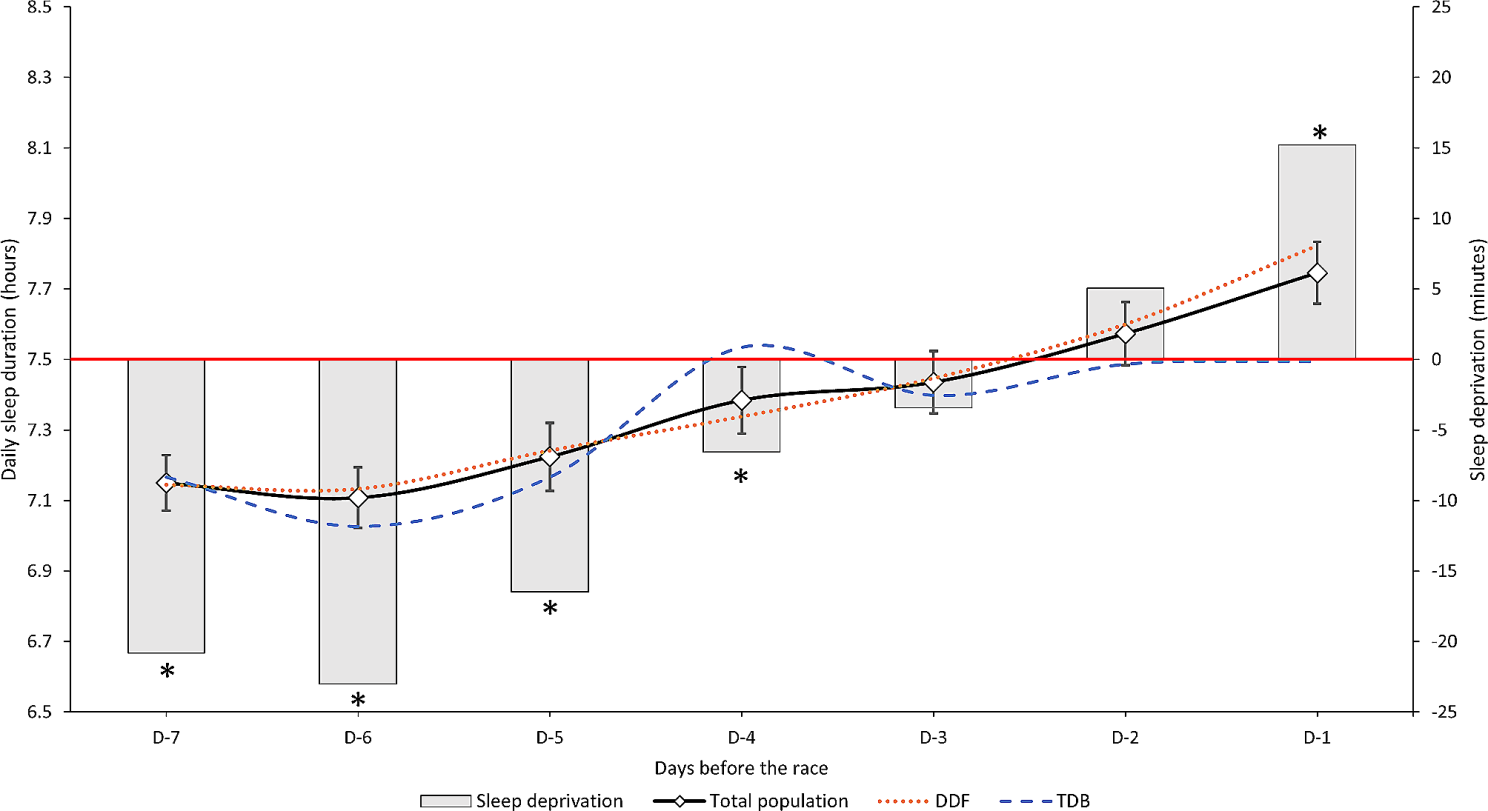
Average daily sleep duration (ADSD) (black line) and sleep deprivation (gray bars) compared to the usual ADSD (horizontal red line) for seven days before the race. The ADSD of the total population is presented with a 95% confidence interval. Orange line = Diagonale Des Fous race (DDF). Blue line = Trail De Bourbon (TDB) race. * = p < 05 refers to a comparison between ADSD for each of the seven days before the race and the usual ADSD

The sleep duration recorded in the sleep diary and sleep debt for the seven days before the race, compared to the usual sleep duration, are presented in Fig. 1. The mean cumulative total sleep debt was − 50, with a median of 0 min, and an interquartile range between − 240 and 210 min. Among runners who believed that they had increased their sleep duration the week before the race, 38.2% had sleep debt during that week. There was no significant difference in the average sleep duration in the week preceding the event between residents and non-residents of Reunion Island in either race (*p* = 0.13). On the night before the race, 28.6% of the participants (26.3% for DDF and 36.0% for TDB) reported sleep disorders-related symptoms (*p* = 0.002). The use of medicines or other alternative therapies (homeopathy, herbal medicine, hypnosis, aromatherapy, and yoga) to help sleep the night before the race was reported by 13.3% of the subjects, without differences between races (*p* = 0.79). However, these values were higher in women than in men (21.2% vs. 12.0%, *p* = 0.001).

### Sleep During the Races

**Fig. 2:**
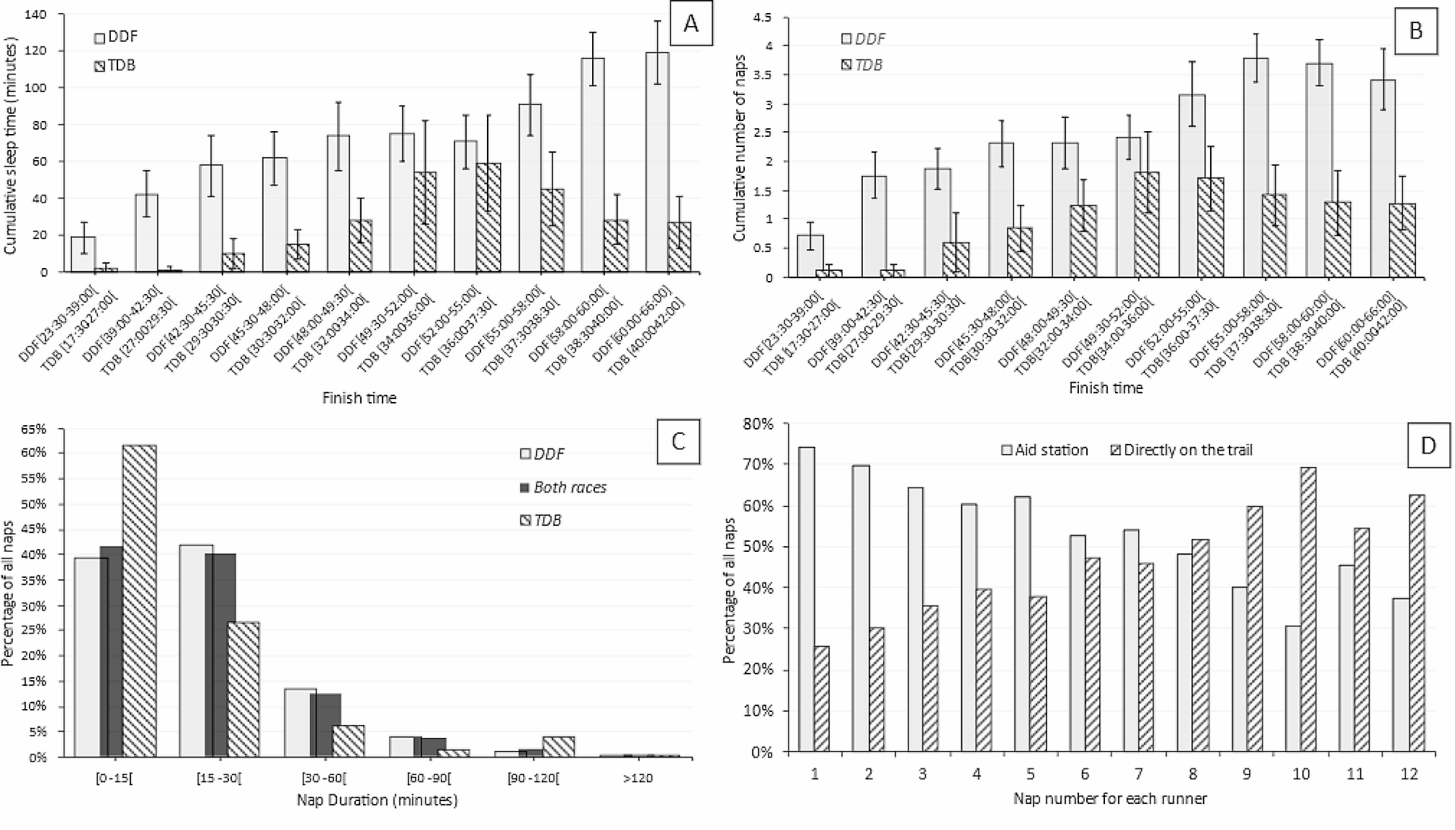
Cumulative sleep time in minutes (panel A) and total number of naps (panel B) during Diagonale des Fous (DDF) and Trail de Bourbon (TDB). Proportions of naps by nap duration on the totality of naps declared by runners of the DDF and TDB (panel C). Proportions of naps by localization where the nap took place, according to the nap number for each runner (panel D)

**Fig. 3:**
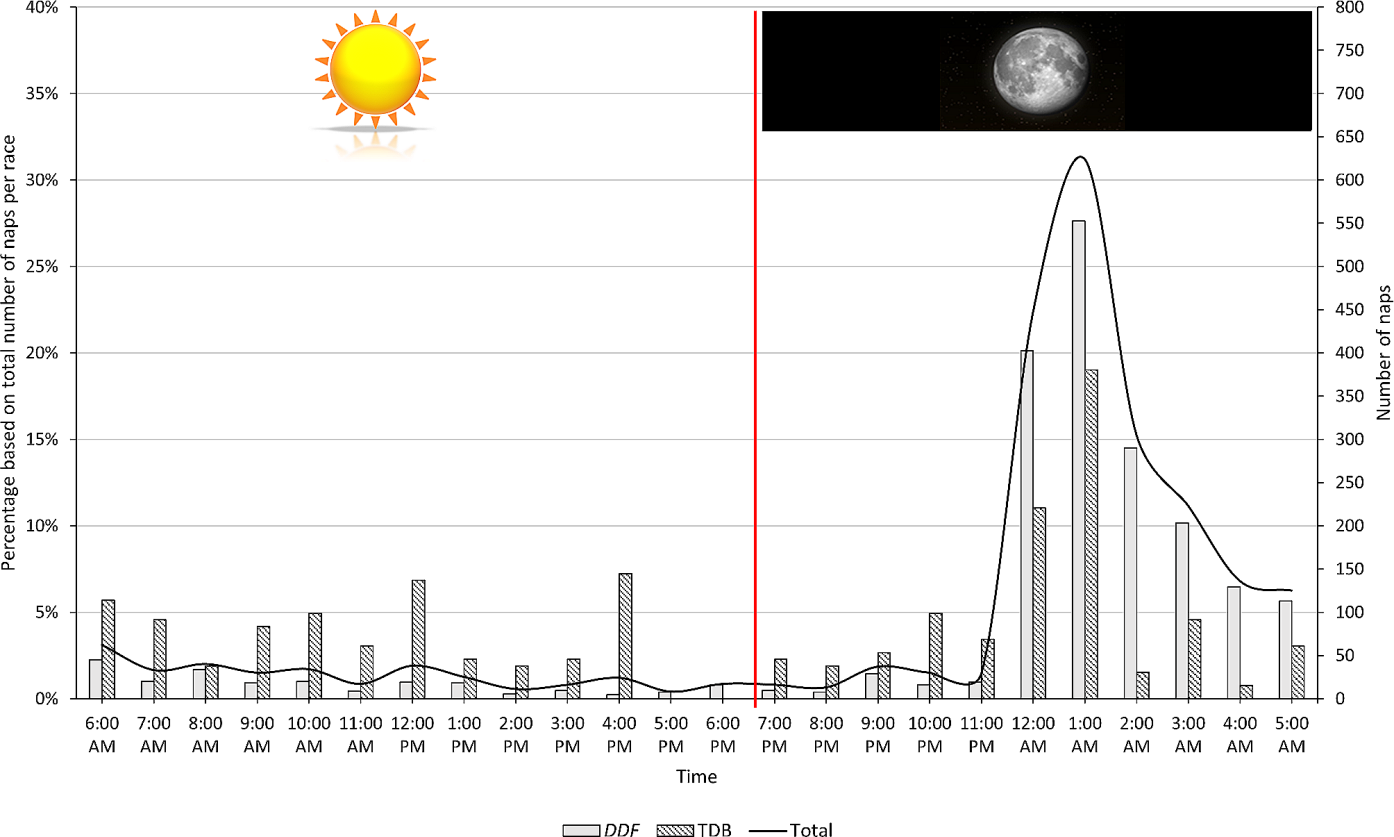
Repartition of number (gray bars) and percentage of naps (black line) over the time of the day. For each hour, the cumulative number of naps over the three days of race is presented. The percentage was calculated based on the total number of naps per race (total number of naps declared for both races:2,340; DDF:2,077; and TDB:263). The vertical red line represents the time of sunset on October 18, 2019, on Reunion Island (6:41 PM)

During the race, 83.8% of DDF runners and 52.9% of TDB runners reported taking at least one nap (*p* < 0.001). Figure 2 presents the characteristics of sleep and naps during the race. The individual cumulative sleep duration during the races was 76.1 ± 80.1 min for DDF and 27.2 ± 44.2 min for TDB (*p* < 0.001). Among runners who spent one night, two nights, or three nights in the race, the cumulative sleep durations were 3.3 ± 8.4 min, 38.5 ± 6 min, and 96.7 ± 83.5 min, respectively. There was a correlation between cumulative sleep duration and race finish time (*r* = 0.44; *p* < 0.0001) in both races (*r* = 0.37; *p* < 0.001 for DDF and *r* = 0.30; *p* < 0.001 for TDB). The cumulative sleep duration was divided into 2.5 ± 2.1 naps for DDF and 1.0 ± 1.3 naps for TDB (*p* < 0.001). Most of the naps (82%) lasted for less than 30 min (Fig. 2c). The location of the naps varied, with 70.3% of the first to fourth naps taken at refreshment/aid stations (on the floor, chair, or bed), and 28.4% directly on the ground near the trail. In contrast, for the 8th to 12th naps, 50% occurred directly on the ground near or on the trail (Fig. 2d). The timing of naps during the race is presented in Fig. 3, indicating that 79.5% of naps occurred between 00:00 AM and 5:00 AM, whereas only 15.2% occurred during the daytime.

### Repercussions of Sleep Deprivation During Races

During the race, 80.1% of runners reported experiencing at least one symptom attributed to sleep deprivation (81.8% during DDF and 74.6% during TDB; p = 0.01). The prevalence of sleep deprivation symptoms varied according to the number of nights spent on the race, as presented in Table 3. The prevalence of “at least one sleep deprivation symptoms” (p < 0.001), “decreased alertness” (p = 0.001), and “hallucination” (p < 0.001) were significantly associated with the number of nights spent in the race. Runners who reported an increase in ADSD had a lower prevalence of falls attributed to sleep deprivation than those who did not (12.3% vs. 17.3%, p = 0.02). However, there were no differences in the finish times (p = 0.37). Prerace sleep strategies were not associated with any other sleep deprivation symptoms.

**Table 3:** Prevalence of symptoms attributed to sleep deprivation for the whole sample and according to the number of nights spent on the race

	Total (*n* = 1,154)	1 night (18)	2 nights (607)	3 nights (527)	*p*
**At least one symptom attributed to sleep deprivation**, n (%)	**924 (80.1)**	**11 (61.1)**	**457 (75.3)**	**454 (86.2)**	**< 0.001**
Decreased alertness	623 (54.0)	8 (44.4)	298 (49.1)	317 (60.2)	0.001
Hallucination	392 (34.0)	1 (5.6)	168 (27.7)	222 (42.1)	< 0.001
Fall	168 (14.6)	1 (5.6)	83 (13.7)	84 (15.9)	0.31
Endangering	112 (9.7)	0	53 (8.7)	59 (11.2)	0.17
Injury	30 (2.6)	0	16 (2.6)	14 (2.7)	1
Other inconveniences (digestive disorders, pain…)	123 (10.7)	2 (11.1)	65 (10.7)	55 (10.4)	0.98

Data are presented as n (percentage). All *p*-values refer to comparisons between one, two, and three nights

### Post-Race and Recovery Period

The average time for runners to return to a normal state of wakefulness without drowsiness after the race was 2.3 ± 2.1 days [2.4 ± 2.2 for DDF and 2.2 ± 1.9 for TDB (*p* = 0.12)]. In this sample, 74.8% of the runners reported a recovery period of less than two days (supplemental 2). Within the first 24 h after the race, ADSD was 9.9 ± 3.3 h [9.8 ± 3.3 h for *DDF* and 10.3 ± 3.4 for *TDB* (*p* = 0.02)]. During the days leading to the recovery of a normal state of wakefulness without drowsiness, runners slept an average of 57.5 ± 141.4 min more than their usual ADSD, with no significant difference between DDF and TDB (*p* = 0.31).

After the race, 22.3% of the runners indicated that the lack of sleep accumulated during the race may have increased the risk of accidents in everyday life, with 21.2% for DDF and 25.7% for TDB (*p* = 0.12). The reported risks included “traffic accidents” (18.5%), “work-related injuries” (3.3%), and “other reasons (falls, injuries)” (2.4%). Drugs or an alternative for sleep resynchronization after the event were used by 3.7% of the runners.

## Discussion

This study represents a significant contribution to the literature, as it investigates sleep patterns in a large cohort of runners (*n* = 1,154) participating in ultramarathon races of varying distances (110 and 165 km). The comprehensive data collected on sleep and ultramarathons set this study apart as the largest of its kind to date. The key findings of this study can be summarized as follows: (i) A notable proportion of runners (58%) reported planning to implement a sleep management strategy either before the race (40%) or during the race (42%). This highlights the importance of sleep in optimizing ultramarathoner performance. (ii) The average cumulative total sleep debt of runners at the start of the race was − 50 min over the seven days preceding the race, compared to their self-reported usual weekly sleep duration. (iii) Despite the high prevalence of napping during the races (77% of runners reported at least one nap), the individual cumulative sleep duration was relatively low, with an average of 76 min for the 165 km race and 27 min for the 110 km race. Naps during races were typically short, with 82% lasting less than 30 min. Moreover, these naps predominantly occurred during the second part of the night, indicating a potential strategy for managing fatigue and enhancing alertness during nighttime running. (iv) 80% of runners reported experiencing at least one symptom attributed to sleep deprivation during a race. However, runners whose ADSD increased in the week leading up to the race had a lower prevalence of falls attributable to sleep deprivation. This suggests that adequate pre-race sleep may have a protective effect against performance-related accidents during the race. vi) Only 22% of the runners indicated that the lack of sleep accumulated during the race might have increased the risk of accidents in everyday life following the race. This finding suggests that ultramarathoners have a relatively low perceived impact of sleep deprivation on their post-race safety.

In our study, we found that only 58% of ultramarathon runners reported having a sleep management strategy before or during their race, which is lower than that reported in previous literature. Previous studies have shown that a higher percentage (79%) of ultramarathon runners are attentive to their sleep on days leading up to an ultramarathon [2]. Specifically, increasing ADSD was the most reported sleep modification (55%), primarily achieved by extending the nighttime sleep duration alone (24%). This finding is consistent with previous research showing that sleep duration is associated with improved physical performance and well-being [23–25] The concept of “banking sleep” before a period of anticipated sleep deprivation has been shown to benefit sports performance. For example, extending nocturnal sleep by 111 min over a 5–7 week period improved various performance measures in basketball players [11]. Similarly, increasing sleep duration by 2 h per night enhances the accuracy of tennis services in healthy individuals [10]. Another study demonstrated that extending sleep by 1.2 h during the six days preceding sleep deprivation improved sustained muscle contraction performance and prevented the degradation of reaction time and occurrence of microsleep episodes [9]. In our study, we observed a lower prevalence of falls among runners who had increased ADSD before the race. Poussel et al. [21] reported that ultramarathon runners who adopted increased sleep time the night before the UTMB® completed the race faster than those who trained for sleep deprivation (*p* = 0.026). However, in our study, we did not find a significant association between sleep modifications before the race and finish time. It is possible that other confounding factors, such as training, fatigue, hydration and feeding management, injuries, and environmental conditions, may have a more significant impact on performance. Additionally, there may be bias in runners’ self-assessments of their sleep duration before the race. Our study revealed that 38.2% of runners who believed they had increased sleep duration had sleep debt according to their sleep diary records. Due to the limited number of responses from non-finishers, we lacked statistical power to evaluate whether banking sleep before a race would reduce the risk of abandonment. Future studies should implement interventional designs to accurately measure ADSD the week before a race and evaluate the true effects of sleep banking on ultramarathon performance. It is also worth noting that while training in a sleep-deprived state was not associated with improved finish times in our population, there have been encouraging results from case studies of male ultra-endurance runners [20].

The results of our study revealed that most ultramarathon runners (77%) reported sleeping during the races. As expected, this proportion and cumulative sleep duration varied depending on race duration and the number of nights spent on the race. For runners who finished their event by spending one night during the race, the cumulative sleep duration was approximately 3 min, whereas for those who spent three nights, it was around 97 min. On the UTMB® (mean race finish time = 39.5 ± 5.1 h), 69–72% of runners reported no sleep [21, 22]. Cumulated sleep duration reported was 0.6 ± 0.7 h for races < 36 h and 1.4 ± 1.5 h for races between 36 and 60 h [2], which is quite comparable to runners who spent three nights on the race during DDF. Athletes competing in 100–149 mile events in the USA (average of 30.9 ± 8.3 h race duration) reported 12.1 ± 50.5 min of total event sleep, much lower than that reported for DDF [18]. Pooling the answers of runners from different races, even at the same distance, can be challenging because of variations in race duration, time limits, trial difficulty, environmental conditions, and participant levels [2, 18]. The duration of the event seems to play a more significant role in assessing sleep needs in ultramarathons, where most runners do not sleep in races lasting one night, but the proportion of sleep increases in races lasting two or three nights, or more than 36 h [18, 19, 21, 22].

Short naps lasting less than 30 min were the most popular sleep strategy reported in our study, which is consistent with previous studies [2, 21, 22]. The timing of these naps aligns with the natural sleepiness promoted by the circadian system during the second half of night [26, 27]. An interesting finding from our study was that the comfort of the napping conditions deteriorated over time. Although 70% of the first naps occurred at aid stations, only 46% of the last naps occurred under the same conditions. This suggests that, as the need for sleep became more pressing, runners were less willing to wait for aid stations and opted to rest in less comfortable areas. The high prevalence of symptoms attributed to sleep deprivation (80%) is concerning. The occurrence of these symptoms was associated with the number of nights spent on the course and duration of the race. The high prevalence of falls and hallucinations during races is particularly worrisome because of the potential for traumatic consequences or life-threatening risks in rugged terrains that are typically found in ultramarathons [28]. Objective measurements of the impact of sleep deprivation on race performance, alertness, and risk taking should be further investigated, as there may be a discrepancy between subjective and objective alertness during prolonged wakefulness [29]. Sleep-restricted individuals are prone to underestimate neurobehavioral impairments [30].

One novel aspect of our study was the assessment of the post-race repercussions of sleep deprivation and analysis of the recovery period, which, to our knowledge, is rarely studied yet crucial in ultramarathon running [13]. Our findings revealed that runners reported recovering a normal state of wakefulness relatively quickly, with 75% stating that it took less than two days which is much lower than previous study where ultramarathoners of shorter race (UTMB®) declared higher hooper index than baseline until 5 days after the race [13]. The recovery period did not differ significantly among the different race formats. Previous research on ∼ 170 km ultramarathons has shown that central fatigue fully recovers within two days after the race [31, 32]. However, it is worth noting that 22% of runners in our study believed that the lack of sleep accumulated during the race could have increased the risk of accidents in their daily lives. This finding aligns with studies on sleep-deprived driving, which have reported an increased risk of accidents among drivers who slept less than four hours the night before [33, 34]. Considering that many runners at Grand Raid are not from Reunion Island and have long flights back home after the race, the effects of sleep deprivation could be further exacerbated by poor quality of recovery during travel. Preliminary results suggest that a recommendation for complete rest for at least two days after an ultramarathon is crucial to promote adequate recovery.

One notable strength of our study was its large sample size (*n* = 1,154), which is the largest among sleep studies conducted in the context of ultramarathons [2, 18, 19, 21]. The study of the post-race recovery period is rare in this discipline, even though the impact of sleep deprivation is significant [13]. However, this study had some limitations. Non-finishers were not included in the analysis because of their small sample size (*n* = 9), which may have underestimated the consequences of sleep deprivation in ultramarathons in our study. Reasons for withdrawal were not determined, which could have influenced the findings. Another limitation is the reliance on self-reported data, which may be subject to reporting and memorization bias. To mitigate these biases, we administered the questionnaire shortly after the race, and the questionnaire itself was detailed by collecting pre-race sleep periods and per-race naps from the sleep diary. However, future studies could employ objective measures, such as actigraphs, portable electroencephalographs, and GPS, to improve the accuracy of sleep duration assessment.

## Conclusions

This study contributes to the understanding of sleep patterns and sleep-related issues in ultramarathon running and provides valuable insights for athletes, coaches, and researchers in the field of sports science and ultra-endurance. These findings highlight the importance of sleep management in optimizing performance and mitigating the negative effects of sleep deprivation during races. This study revealed the potential benefits of increasing sleep duration before the race, as it was associated with a lower prevalence of falls during the race. Short napping, particularly during the second half of the night, has emerged as a popular approach for managing sleep deprivation in races. However, this study also highlights the high prevalence of symptoms attributed to sleep deprivation and the potential risks associated with impaired alertness during prolonged wakefulness. Post-race recovery was relatively quick, with most runners returning to a normal state of wakefulness within two days. Nevertheless, a significant proportion of runners perceived a potential increase in the risk of accidents in everyday life due to accumulated sleep deprivation during the race, emphasizing the need for proper recovery after ultramarathons. Future studies should further investigate the impact of sleep management strategies on race performance and explore objective measurements of sleep duration. Additionally, interventions and recommendations for adequate rest and recovery after ultramarathons should be developed to enhance athletes’ well-being and safety.

### Electronic Supplementary Material

Below is the link to the electronic supplementary material.

**Supplemental 1**. Sleep and Ultramarathon Questionnaire (English version).

**Supplemental 2**. Proportion of runners having recovered a state of wakefulness without drowsiness after the race.

## Data Availability

The data collected for this study are available upon request, supporting the principles of data sharing and transparency in scientific research.

## Abbreviations

UTMB®: Ultra-Trail du Mont Blanc®
DDF: La Diagonale des Fous
TDB: Le Trail de Bourbon
ADSD: Average daily sleep duration
