# Supplementary material for: Sleep and Ultramarathon: Exploring Patterns, Strategies, and Repercussions of 1,154 Mountain Ultramarathons Finishers
Source: Sports Med Open. 2024 Apr 8;10:34. doi: 10.1186/s40798-024-00704-w (PMC11001838; doi:10.1186/s40798-024-00704-w)
Supplement: Supplementary file 1 — Supplemental 1. Sleep and Ultramarathon Questionnaire (English version). [file 40798_2024_704_MOESM1_ESM.pdf]

ORIGINAL ARTICLE: SPORTS MEDICINE-OPEN

Title: SLEEP AND ULTRAMARATHON: EXPLORING PATTERNS, STRATEGIES, AND REPERCUSSIONS OF 1,154 MOUNTAIN ULTRAMARATHONS FINISHERS

SHORT TITLE: Sleep and ultramarathon

AUTHORS AND AFFILIATIONS DETAILS

Anthony Kishi [1], Guillaume Y Millet[2,3], Matthieu Desplan[4], Bruno Lemarchand[1], Bouscaren Nicolas[2,6]

1- Unité Fonctionnelle de Médecine du Sport, CHU de la Réunion, Site Hôpital de Saint-Pierre, BP 350, 97448 Saint-Pierre, France
2- Univ Lyon, UJM-Saint-Etienne, Inter-university Laboratory of Human Movement Biology, EA 7424, F-42023, Saint-Etienne, France
3- Institut Universitaire de France (IUF)
4- Be Sports Clinic, Centre Médical Médimarien, 21 rue Marcel Marien 1030 Schaerbeek, Belgique
5- Unité Fonctionnelle de Médecine du Sport, CHU de la Réunion, Site Hôpital de Saint-Pierre, BP 350, 97448 Saint-Pierre, France
6- Inserm CIC1410, Service de santé publique et soutien à la recherche, CHU Réunion, Saint Pierre, France.

CORRESPONDING AUTHOR

Dr BOUSCAREN Nicolas (MD)
INSERM CIC 1410, Service de santé Publique et soutien à la recherche, CHU Réunion
Tel : 02 62 71 98 30   ORCID ID : 0000-0001-9853-098X
Email : n.bouscaren@gmail.com / nicolas.bouscaren@chu-reunion.fr

Sleep and Ultramarathon Questionnaire

*Mandatory questions

How old are you? *
_ _ Year Old

You are: *
- A man
- A women

You participated: *
- Trail de Bourbon 2018
- Diagonal des fous 2018

Are you finisher of the race in which you participated?
- Yes
- No

What was your time of arrival or dropout (depending on the situation)?
__h__min__sec

Your Trail practice

On average, for how long did you train every week in preparation for this race *
__h__min

How many years have you practiced trail running?
__ years

Was The « Diagonale des Fous » or « Trail de Bourbon » your first ultra-trail? (Mountain race > 80km according to the FFA - French Federation of Athletics - definition)
- Yes
- No

How many other ultra-trails have you doned/finished? *

The acquired experience with these other ultratrails helped manage your sleep better during this last race:
- Yes
- No
- I have no idea

Most of the time, how many hours a day do you sleep (including naps) *

Most of the time, what time do you get you *

Most of the time, what time do you go to bed *

Ideally, how many hours of sleep a day would you need to feel good (including naps) *

If you could follow your sleep rhythm (the one that pleases you the most), at what time you would get up?

If you could follow your sleep rhythm (the one that pleases you the most), at what time would you go to bed?

Do you work at night? *

- Yes
- No

You would define yourself as *

- A morning person
- A Night owl
- Neither

I think I'm affected by: (Zero or several possible answers)

- sleep apnea
- insomnia
- depression

You have already taken or recently used one of the following treatments: (0 or several possible answers)

- An antidepressant
- A sleeping pill
- An equipment of ventilation for sleep

General sleep strategy for the race

You have already taken or recently used one of the following treatments: (0 or several possible answers)

- Yes
- No

Have you decided to modify your sleep pattern (before, during, and after the race), including whether you chose not to sleep?

- For the period before the race
- For the race itself
- For the period after the race
- I didn't develop a strategy related to the sleep

If you had developed one or several strategies for sleep, you respected them (zero or several possible answers).

- For the period before the race
- For the race itself
- For the period after the race

If you had developed one or several strategies for sleep, you respected them (Zero or several possible answers):

- Own experience
- Information found on the Internet
- Advice from friends or other runners
- Coach advice
- Other

Before the departure, concerning my sleep strategy during the race : *

- I had planned not to sleep at all
- I planned all of my sleep periods.
- I had planned to sleep when I would feel the need, no matter the place
- I had planned to sleep when I would feel the need, preferably at the (refreshment) supply point
- I had not thought of this question at all

Your sleep before the race

This part of the questionnaire concerns your sleep before the race, particularly the week before departure.

The week before the race, what rhythm (s) represented (s) your modification (or not) of sleep? (Several possible answers)

- I increased my duration of sleep at night (earlier bedtime and getting up late
- I increased my daytime sleep by implementing nap
- I kept my duration of sleep, but I moved my schedule around: sleeping later and getting up late
- I kept my duration of sleep, but I moved my schedule: sleeping earlier and raising earlier
- I kept my duration of sleep, but I split my sleeping time
- I have not changed my sleeping rhythm

I have undergone a period of involuntary and unusual sleep deprivation (long-haul flight, jet lag, insomnia due to stress, and other reasons for sleep deprivation):

- Yes
- No

If yes, why were you deprived of sleep?

- Long haul flight
- Jet lag
- Insomnia bound to the stress

From memory and on average, fill in the number of hours you slept a day (night + naps) the week before departure:

Move the horizontal cursor as needed to select the corresponding duration if it doesn't appear

- ⇨ 1-12 hours of sleep
- ⇨ For the days: Thursday, October 11th Friday, October 12th Saturday, October 13th Sunday, October 14th, Monday, October 15th Tuesday, October 16th, Wednesday, October 17th Thursday, October 18th

Did you have difficulties sleeping the night before the departure of the race *

- Yes
- No

Specify the quality of the last night of sleep before the departure : *

- Very Bad
- Bad
- Average
- Good
- Very good

How long do you think you have slept (at night + including naps) over the 24 hours before your departure?

From 10:00 pm on October 17th to 10:00 pm on October 18th for the « Diagonale des Fous » or from 9:00 pm on October 18th to 9:00 pm on October 19th for the « Trail de Bourbon."

Have you used medicines or other alternatives (herbal teas, relaxation therapy, homeopathy, alternative treatments, herbal medicine, etc.) before the race to help you sleep

- Yes
- No

If yes, specify which

- Medicine
- herbal teas
- relaxation therapy
- Homeopathy
- Hypnosis
- Yoga
- Herbal medicine
- Aromatherapy

Concerning my physical condition before the departure of the race, I felt *

Mark the figure which corresponds best to your physical condition: this scale goes from 0= very tired to 5= very in shape

Your sleep during the race

This part of the questionnaire focuses on sleep management throughout the race.

During the race (several possible answers) *

- I've had moments when I was less vigilance or even slumber
- I think I've had hallucinations
- I think I have fallen or almost fallen because of a lack of vigilance
- I think that the lack of sleep could have put me in danger
- I think that one of my wounds is due (at least partially) to the lack of sleep
- I've had other inconveniences I attribute (at least partially) to the lack or the mismanagement of my sleep
- (if yes, specify in the « other" section)

During the race, how many periods of sleep did you have *

- I didn't sleep (0 nap)
- __ naps (1-20)

Can you estimate your total duration of sleep throughout the race *
__h__min__Sec

Tick the (approximate) time of your naps during the race
Move the horizontal cursor to reveal schedules
- ➪ 0:00 am to 11:00 pm
- ➪ Thursday, October 18th to Sunday, October 21st

How long have your naps been?
Fill in duration for each nap.
- 0 -15 min
- 15 – 30 min
- 30 – 60 min
- an hour to an hour and a half
- An hour an a half to 2 hours
- 2 to 3 hours
- > 3 hours
- ➪ For Nap n°1 to n°xx

Under what conditions did you take these naps?
- On the path or at the edge of
- the path on the floor
- In a refreshment/supply point on
- a bed or an armchair
- In a refreshment
- point on the floor
- Other
- ➪ For Nap n°1 to n°xx

I have the impression that these periods of sleep were helpful for me to feel more careful during the race:
By « careful," we mean that you felt more awake, with a better lucidity and better reflex
- Yes
- No
- I don't know

After the race

For how long did you sleep (naps included) the first 24 hours after your arrival or dropout *
Example: if you arrived (or abandoned) at 3:30 pm on Saturday, the considered period extends to 3:30 pm the next day (on Sunday)
__h__min__sec

After the race, I found a state of usual awakening without slumber at the end of: *
- From the 1st day
- __ days

On this first day with an unusual slumber, I consider that my duration of sleep a day was : *
__h__min__sec

In the following days, when my state of usual awakening was recovered, I considered that my duration of sleep per day was on average:
- Yes
- No

I think that the lack of sleep accumulated during the race may have increased the risk of health problems after the race (while driving, during other situations of endangerment):
- Yes
- No

If yes, specify which risk (s) was/were increased for you.
- Driving
- Accident at work
- Other

Have you used medicines or other alternatives (herbal teas, relaxation therapy, homeopathy,

Alternative treatments, herbal medicines, etc. ..) After the race, to resynchronize sleep or help you sleep?

- Yes
- No

If yes, specify which :

- Medicine
- Homeopathy
- Herbal medicine
- herbal teas
- Hypnosis
- Aromatherapy
- relaxation therapy
- Yoga

Compared with other subjects such as food or hydration, you think that the question of sleep in the ultra trail is something which is:

- Unimportant
- Important
- Insignificant
- Very important

Your Sleep profile

Most of the time, how many hours a day do you sleep (including naps) *

__h__min_sec

Most of the time, what time do you get you *

__h__min_sec

Most of the time, what time do you go to bed *

__h__min_sec

Ideally, how many hours of sleep a day would you need to feel good (including naps) *

__h__min_sec

If you could follow your sleep rhythm (the one that pleases you the most), at what time would you get up?

__h__min_sec

If you could follow your sleep rhythm (the one that pleases you the most), at what time would you go to bed?

__h__min_sec

Do you work at night? *

- Yes
- No

You would define yourself as *

- A morning person
- A night owl
- Neither

I think I'm affected by: (Zero or several possible answers)

- sleep apnea
- insomnia
- depression

You have already taken or recently used one of the following treatments: (0 or several possible answers)

- An antidepressant
- An equipment of ventilation for sleep
- A sleeping pill
