# Supplementary material for: Sleep and Ultramarathon: Exploring Patterns, Strategies, and Repercussions of 1,154 Mountain Ultramarathons Finishers
Source: Sports Med Open. 2024 Apr 8;10:34. doi: 10.1186/s40798-024-00704-w (PMC11001838; doi:10.1186/s40798-024-00704-w)
Supplement: Supplementary file 2 — Supplemental 2. Proportion of runners having recovered a state of wakefulness without drowsiness after the race. [file 40798_2024_704_MOESM2_ESM.pdf]

ORIGINAL ARTICLE: SPORTS MEDICINE-OPEN

Title: SLEEP AND ULTRAMARATHON: EXPLORING PATTERNS, STRATEGIES, AND REPERCUSSIONS OF 1,154 MOUNTAIN ULTRAMARATHONS FINISHERS

SHORT TITLE: Sleep and ultramarathon

AUTHORS AND AFFILIATIONS DETAILS

Anthony Kishi [1], Guillaume Y Millet[2,3], Matthieu Desplan[4], Bruno Lemarchand[1], Bouscaren Nicolas[2,6]

1- Unité Fonctionnelle de Médecine du Sport, CHU de la Réunion, Site Hôpital de Saint-Pierre, BP 350, 97448 Saint-Pierre, France
2- Univ Lyon, UJM-Saint-Etienne, Inter-university Laboratory of Human Movement Biology, EA 7424, F-42023, Saint-Etienne, France
3- Institut Universitaire de France (IUF)
4- Be Sports Clinic, Centre Médical Médimarien, 21 rue Marcel Marien 1030 Schaerbeek, Belgique
5- Unité Fonctionnelle de Médecine du Sport, CHU de la Réunion, Site Hôpital de Saint-Pierre, BP 350, 97448 Saint-Pierre, France
6- Inserm CIC1410, Service de santé publique et soutien à la recherche, CHU Réunion, Saint Pierre, France.

CORRESPONDING AUTHOR

Dr BOUSCAREN Nicolas (MD)
INSERM CIC 1410, Service de santé Publique et soutien à la recherche, CHU Réunion
Tel : 02 62 71 98 30   ORCID ID : 0000-0001-9853-098X
Email : n.bouscaren@gmail.com / nicolas.bouscaren@chu-reunion.fr

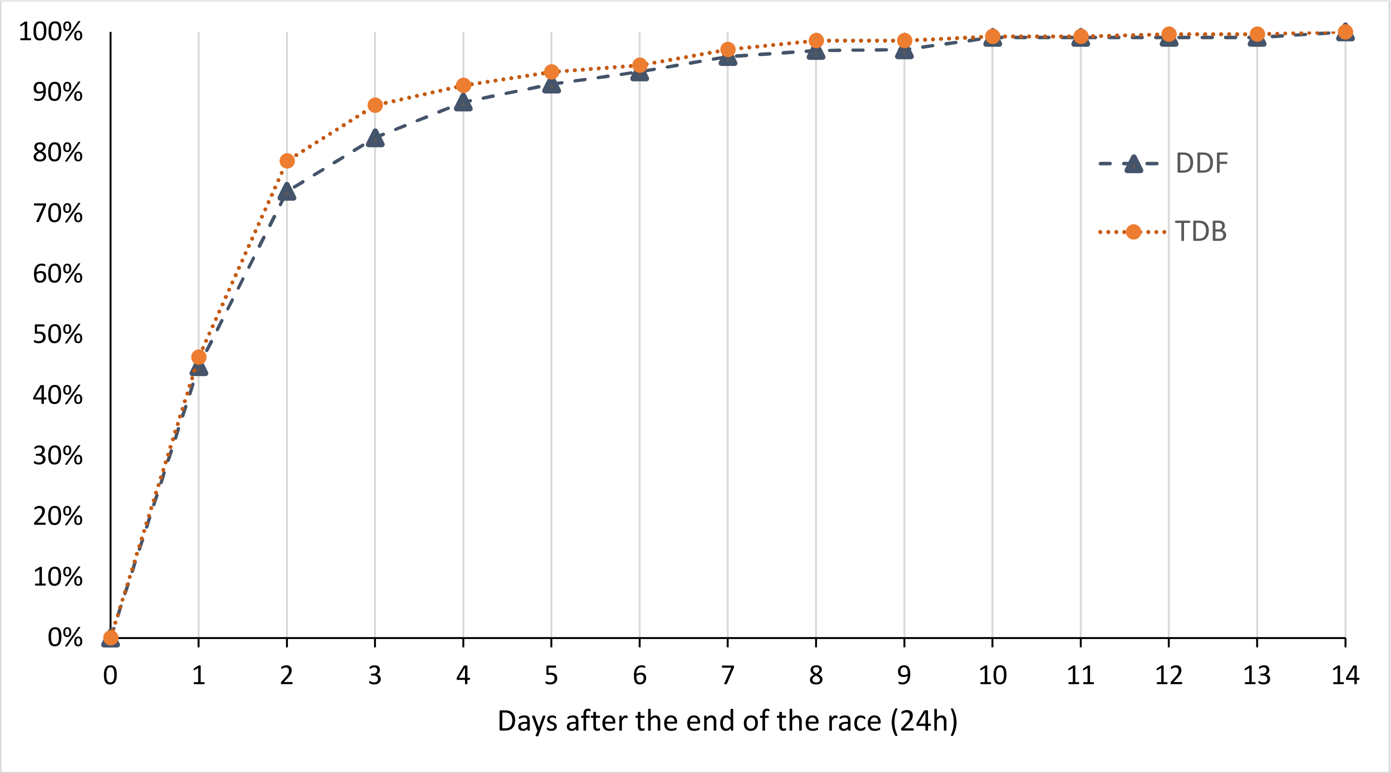

Supplemental 2: Proportion of runners having recovered a state of wakefulness without drowsiness after the race**.** The percentages were calculated as the total number of runners for each event.
